# The Effect of Pattern Addition on the Mechanical Properties of 3D-Printed Parts

**DOI:** 10.3390/polym17243327

**Published:** 2025-12-17

**Authors:** Nergizhan Anaç, Oğuz Koçar

**Affiliations:** 1 Department of Mechanical Engineering, Faculty of Engineering, Zonguldak Bülent Ecevit University, Zonguldak 67100, Türkiye; oguz.kocar@yahoo.com.tr; 2 Zonguldak Teknopark, Çınartepe Mahallesi Adnan Menderes Caddesi, No: 91C/1, Zonguldak 67040, Türkiye

**Keywords:** additive manufacturing, 3D printing, multi-material, surface pattern, mechanical properties

## Abstract

Additive manufacturing is a suitable method for multi-material production, as it offers flexibility in structural design and enables layer-by-layer fabrication of materials. However, the different chemical structures and formulations of the materials used may affect the mechanical integrity of the part. In nature, there are many patterned structures that inspire the design of multi-material additive manufacturing components. Integrating the harmony and advantages of these natural structures into the manufacturing process will significantly contribute to human development. This study presents a novel manufacturing approach for using existing natural or artificially produced pattern forms in the development of composite materials. In this aim, patterned parts composed of multiple materials were produced using a 3D printer with combinations of PLA Plus, PLA CF, and PLA GF. Mechanical tests were conducted on the produced parts, and their fracture surfaces were examined. In patterned specimens, tensile strength decreased compared to reference (non-patterned) specimens. In the PLA Plus–PLA Plus and PLA Plus–PLA CF combinations, tensile strength generally decreased in samples with three patterns, while the greatest reduction in tensile strength occurred in the PLA Plus–PLA GF patterned specimens. The highest bending forces were obtained in single- and five-pattern samples with PLA Plus–PLA Plus and PLA Plus–PLA CF combinations, as well as in five-pattern samples with the PLA Plus–PLA GF combination. The results indicate that the presence and number of patterns are important factors influencing the mechanical properties of the specimens.

## 1. Introduction

Multi-material 3D printing involves the process of depositing multiple materials layer by layer on a single platform, resulting in the fabrication of a new multi-material structure [1]. In traditional manufacturing, obtaining a multi-material part is costly and may require advanced expertise and specialized equipment [2]. Advanced 3D printers are capable of simultaneously printing more than one material through dual-extrusion systems. A wide range of multi-material combinations can be produced, including metal–metal, metal–ceramic, polymer–polymer, and others. Multi-material structures can be manufactured by considering the compatibility of the constituent materials, the geometry of the structure, printing process parameters, and environmental conditions [3]. Additive manufacturing is regarded as a transformative technology for multi-material production due to its geometric flexibility, the nature of its fabrication process, and its environmentally friendly characteristics [4]. However, the mechanical properties of Fused Deposition Modeling (FDM)-based products may be weaker compared to those produced using conventional manufacturing methods [5].

Lopez and Ahmad [6] produced sandwich-structured specimens in their multi-material studies using fused deposition modeling (FDM) technology to combine different plastic materials such as polylactic acid (PLA), acrylonitrile butadiene styrene (ABS), and high-impact polystyrene (HIPS). Galatas et al. [7] printed multi-material specimens using various combinations of ABS and PLA in a 3D printer. They reported that the positioning of the materials used in multi-material fabrication significantly affects the strength of the printed part and the overall performance of the process [5,8].

Experimental observations have shown that the chemical structures and formulations of the thermoplastics used in multi-material 3D printing present significant challenges. The need for different printing temperatures and printing speeds for different materials limits the feasibility of the printing process [9]. Different polymer materials possess distinct chemical structures. Therefore, combining dissimilar materials may lead to problems due to mismatches in their coefficients of thermal expansion. As a result, conditions such as differential shrinkage during cooling can ultimately cause deformation and reduced dimensional stability of the printed components. In addition, the expected mechanical integrity at the interfaces between various printed materials is reduced due to chemical incompatibility or weak chemical bonding. The interfacial characteristics of the different materials that form a 3D multi-material structure exhibit lower bonding strength compared to the overall strength of the material. This situation arises from the discontinuous nature of the FDM process during multi-material fabrication [10].

Nevertheless, the production of complex geometries using additive manufacturing with multi-material systems provides additional functionality, environmental adaptability, and enhanced mechanical properties to the final products [11]. Nature contains numerous structures that inspire the design of multi-material additive manufacturing components and the development of advanced engineering materials. These structures are fundamentally based on the forms or behaviors of plants and animals. Gu et al. [12] investigated the impact performance of multi-material designs produced via 3D printing that mimic the architecture (microstructure) of natural seashells. Their study indicated that incorporating seashell-inspired architecture and damage mechanisms into the design of protective equipment—such as helmets and body armor—may be feasible. In addition, various armor designs produced using 3D printing have been developed by drawing inspiration from biological systems such as fish scales, crocodile skin, and turtle shells, with the aim of improving energy dissipation and impact resistance [13,14,15,16].

Structures in nature have an inherent order and, as a whole, are much stronger than the individual components that constitute them. The biomaterials found in organisms that have evolved over millions of years generally exhibit the high strength and toughness that contemporary engineering materials strive to achieve [12,17,18]. In the literature, numerous studies based on this approach have been conducted in various fields, and research in this direction is still ongoing. Designs adapted from bird claws have been used for various gripping applications [19]. Spider silk has been compared with synthetic fibers such as nylon or Kevlar in terms of strength [17]. Silent and high-speed train designs have been developed inspired by the beak of a bird. For next-generation armor design, researchers continue to draw biological inspiration from fish, crocodiles, armadillos and seashells. In all of these studies, researchers have focused primarily on the known and predictable properties of natural structures. Designing by copying the behaviors or forms observed in nature and proposing them as solutions in engineering processes has now become commonplace [20].

In the 1950s, Otto Schmitt introduced the concept of biomimetics into the literature [21]. The first model explaining how patterns found in animals are formed was proposed by Alan Turing in 1952. Later, in 1997, Janine Benyus popularized the term biomimicry in her book [22]. Since then, research demonstrating the use of biology and nature in creative solutions—such as utilizing information derived from animal patterns—has continued to grow. However, measurement and analysis methods for patterns are generally insufficient and, in most cases, pattern characteristics are described based on human perception of their appearance [23].

In reality, since patterns do not possess clearly defined boundaries, they require more detailed evaluation [24]. Nevertheless, among the countless existing natural patterns, the potential for creating new composite material designs remains remarkably high. In their study, Masrouri et al. [25] argued that considering the patterns found on animal skin is meaningful for composite material design. They emphasized that understanding a pattern that serves a specific purpose can support the composite manufacturing process. They adopted the idea that contrasting colors observed in patterns may be interpreted as the matrix and reinforcement materials that constitute a composite. It is known that the biological structures that inspire material design are not monolithic or continuous in form [26]; rather, they are generally multi-layered. The presence of multiple materials in natural organisms provides excellent balance and performance by functionally grading their advantages [26,27]. Approaches in multi-material additive manufacturing enhance design capabilities for multifunctional and functionally graded materials and structures [28]. This situation strongly encourages researchers to develop new products by taking advantage of the interaction between natural structures and laboratory-produced composite materials. During the development process, it is recommended to combine materials such as acrylonitrile butadiene styrene (ABS), polylactic acid (PLA), and polyether ether ketone (PEEK) as matrix materials, together with reinforcements such as carbon fiber, in order to obtain different properties [29]. In light of these considerations, it is understood that the use of 3D printing is essential in multi-material manufacturing to modify the final component properties.

However, in the literature, no manufacturing approach has been encountered regarding the use of patterns found in natural organisms in composite material production or the incorporation of multi-material patterned structures into engineering solutions. It is not easy to transfer every piece of knowledge gained from nature into industrial applications. A multidimensional perspective is required in order for the concept of pattern in nature to be utilized in engineering practice.

First, it is essential to understand the biological purpose of the patterns specific to organisms. Then, the fundamental process steps such as selecting the pattern for multi-material patterned production, determining its dimensions, visualizing it, converting it into a digital form, selecting the materials, and positioning the pattern within the structure are carried out. Finally, an appropriate manufacturing method must be chosen. At this stage, the difficulties that may arise in producing multi-material patterned structures using conventional methods can be overcome through the use of innovative manufacturing tools such as 3D printers.

Accordingly, in this study, the flexibility offered by 3D printers in terms of material and design diversity in multi-material production was utilized. The aim was to demonstrate the potential of using naturally occurring or artificially generated patterns as a new approach in composite material production.

In this study, fully dense (100% infill ratio) multi-material specimens were produced using a 3D printer with different combinations of PLA Plus, PLA CF, and PLA GF materials (PLA Plus–PLA Plus, PLA Plus–PLA CF, and PLA Plus–PLA GF). The multi-material structures consist of two main components: a pattern and the substrate on which the pattern is placed (base material). A newly designed pattern geometry developed by the authors was used as the patterned element. The pattern form was arranged on the specimens sequentially in sets of 1 (single), 3, and 5. Tensile and 3-point bending tests were conducted to determine the mechanical properties of the samples. The study aimed to examine the effect of incorporating patterns into 3D-printed multi-material composites on their mechanical performance. The work focused on understanding the strength relationship between specimens with discontinuous surfaces (patterned) and those with continuous surfaces. It is believed that the outcomes of this study will support the consideration of pattern-based reinforcements (pattern addition) as a viable solution in the development of mechanisms for manufacturing composite materials.

## 2. Materials and Methods

### 2.1. Properties of Materials

In the study, three different types of filament materials with a diameter of 1.75 mm, produced by the brand Esun (Shenzhen, China)—PLA Plus, carbon fiber-reinforced PLA (PLA CF), and glass fiber-reinforced PLA (PLA GF)—were used. PLA, by its nature, is an environmentally friendly material that does not harm human health, has no toxic effects, and helps reduce the carbon footprint. PLA Plus is the strengthened version of PLA filament. In PLA Plus, PLA’s weak points—such as moisture absorption and brittleness—have been improved. Additionally, its toughness and interlayer adhesion are better than those of standard PLA. PLA CF is produced by reinforcing PLA with short chopped carbon fibers. The addition of carbon fibers gives the filament a matte color while improving its bending and tensile strength. Glass fiber-reinforced PLA (PLA GF) is produced by adding 16% glass fiber to PLA, resulting in improved stiffness and impact resistance. PLA Plus filaments are black and white in color, carbon fiber-reinforced PLA (PLA CF) filaments are black, and glass fiber-reinforced PLA (PLA GF) filaments are white. In choosing filament colors, black and white were intentionally preferred to ensure a clear distinction between the pattern and the base material. The color contrast between black and white allows the elements to be fully defined and visually balanced, which guided this choice [30]. The mechanical properties of the filament materials used in the study are presented in Table 1.

### 2.2. Three-Dimensional Printing Parameters and Tests

**Figure 1:**
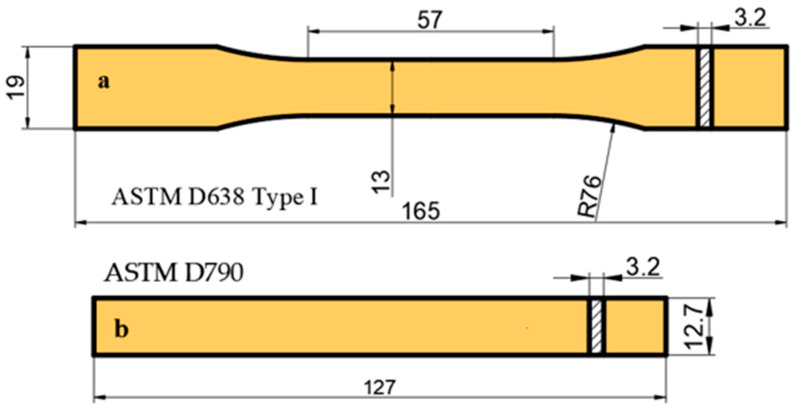
Sample dimensions of tensile (**a**) and bending (**b**) (dimensions are in mm).

In the experiments, samples (reference and patterned) were produced from PLA Plus-PLA Plus, PLA Plus-PLA CF and PLA Plus-PLA GF material pairs. The samples were printed using a Bambu Lab P1S Combo printer (Bambu Lab, Shenzhen, China) operating on the fused deposition modeling (FDM) principle. For the evaluation of the mechanical properties of the material pairs, the reference samples used in the tensile and bending tests were reprinted each time and measured accordingly. To determine the mechanical properties of the samples, they were prepared in accordance with the ASTM D638 Type I tensile standard [34] and the ASTM D790 bending standard [35]. The drawings required for printing the samples were prepared in the “SolidWorks 2024” program and converted into STL format. They were then imported into the “Bambu Studio 2.3.1 version” software, where the processing parameters were assigned and slicing was performed. Figure 1 presents the dimensions of the tensile and bending specimens. Tensile tests were conducted at a test speed of 1 mm/min, and bending tests at 5 mm/min (at room temperature). A WDW-5 model universal testing machine with a capacity of 5 kN was used for all tests, each performed with four repetitions. PLA Plus and PLA CF specimens were printed at a nozzle temperature of 215 °C, a bed temperature of 55 °C, and a printing speed of 100 mm/s. For PLA GF specimens, the printing parameters were set as follows: nozzle temperature 220 °C, bed temperature 60 °C, printing speed 80 mm/s, and printing orientation XYZ. All samples were printed with a layer height of 0.2 mm and a 100% infill ratio. For each material, four tensile and four bending specimens were produced, and the average strength values were calculated from these samples. Hardness measurements were performed according to ASTM D2240 [36] using a Loyka Shore-D durometer (Shenzen Yibai Network Technology Co., Ltd., Shenzhen, China). For each sample, hardness was measured at ten different points, and the average value was calculated.

An FEI-Quanta FEG 250 system scanning electron microscope (SEM) (Thermo Fisher Scientific, Waltham, MA, USA) was used to obtain images of the samples, which were coated with a thin layer of gold and palladium prior to examination.

### 2.3. Experimental Design and Pattern Dimensions

**Figure 2:**
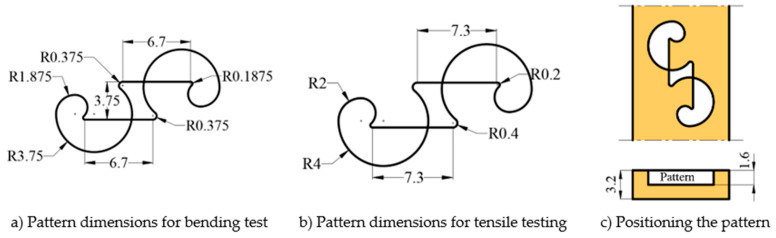
Pattern Dimensions Designed for Bending and Tensile Test Specimens.

**Figure 3:**
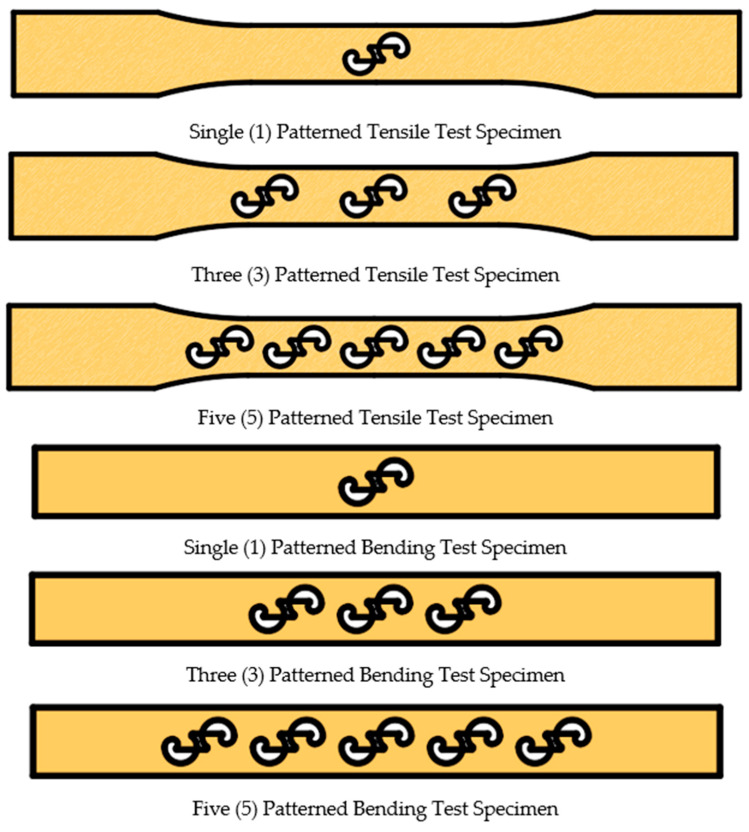
The placement of the patterns on the surfaces of the bending and tensile test specimens.

The surface pattern used on the specimens in this study was designed by the authors, and a design registration certificate was obtained for it [37]. While designing the specimen, the primary reason for not selecting a pattern found in nature was the intention to use a form with clearly defined boundaries. Figure 2a,b present the dimensions of the patterns placed on the bending and tensile specimens. The pattern dimensions were scaled to ensure equal positioning on both bending and tensile samples. The specimens were printed with a thickness of 3.2 mm. The printing of the patterns began at half the specimen height and ended at the outer surface of the part. The pattern height is 1.6 mm. Figure 2c shows the dimensional representation of the specimen and the pattern (in mm). The patterns were positioned sequentially on the tensile and bending samples with 1, 3, and 5 repetitions. Figure 3 shows the placement of the patterns on the specimens.

The material types were defined as PLA Plus (PP), PLA GF (GF), PLA CF (CF), and reference (Ref). Since two different colors were used for the base material and the pattern; the colors were designated as black (B) and white (W). Additionally, a number was added to the end of these codes to indicate the number of pattern repetitions. Table 2 presents an example of the coding scheme used to represent the samples.

## 3. Findings and Results

### 3.1. Mechanical Properties of Reference Samples

**Figure 4:**
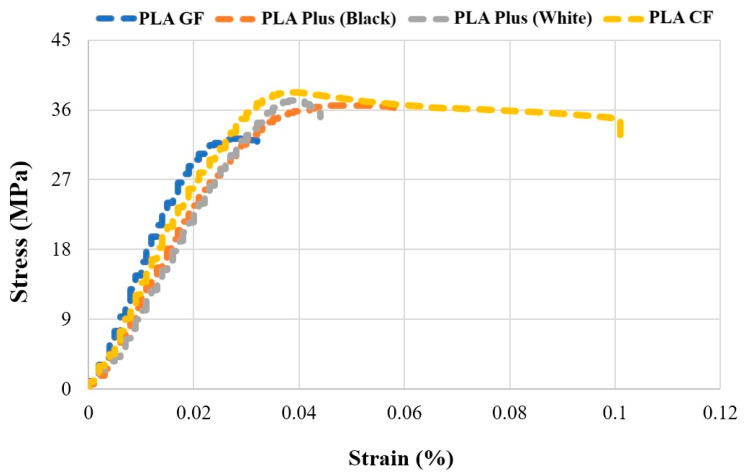
Stress–Strain Curves of Reference Specimens.

As reference samples, the mechanical properties (hardness, tensile strength, and bending force) of PLA Plus (in both white and black), PLA CF, and PLA GF without any pattern were determined. The stress–strain diagram obtained from the tensile tests is presented in Figure 4, and the tensile stress, percentage elongation, elastic modulus, and bending force values are provided in Table 3. The UTS values obtained from the tensile tests were determined as 36.63 ± 0.4 MPa for black PLA Plus, 30.8 ± 1.0 MPa for white PLA Plus, 37.83 ± 0.4 MPa for PLA CF, and 32.57 ± 1.1 MPa for PLA GF. The highest tensile strength and the highest percentage elongation (9.23%) were obtained from PLA CF. The addition of carbon fiber has a positive effect on the ductility and mechanical properties of PLA and PLA-based materials. The elastic modulus values of PLA CF (1077.06 MPa) and PLA GF (1077.30 MPa) were found to be close to each other. When the bending force values were examined, the highest value was obtained as 111.4 ± 1.3 N for PLA CF. The Shore D hardness values were measured as 82.6 ± 1.8 for PLA Plus, 84.9 ± 2.0 for PLA CF, and 82.1 ± 0.5 for PLA GF. In addition, the mechanical properties of PLA Plus materials varied depending on the color. Studies in the literature on PLA and ABS also report that mechanical properties change depending on color [38,39,40].

### 3.2. Effect of Pattern on Print Quality

**Figure 5:**
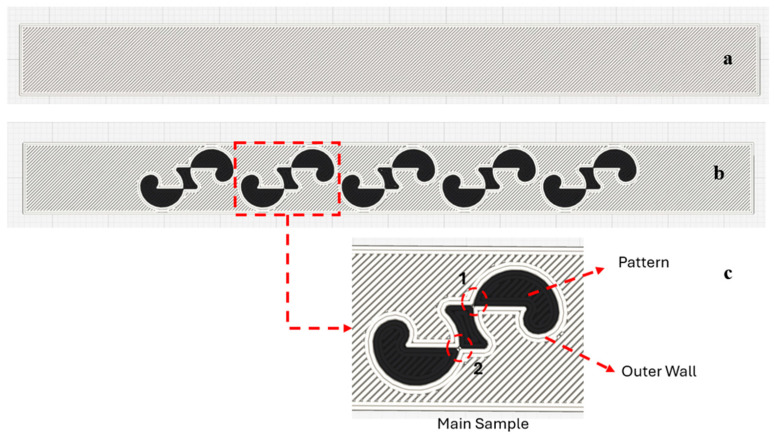
Image of the sample after slicing. (**a**) Unpatterned sample. (**b**) Patterned sample (**c**) Patterned walls and narrow regions (1 and 2).

Figure 5 shows the sliced views of the reference specimen (a) and the patterned specimen (b). In Figure 5c, the walls formed at the transition between the pattern and the base material are illustrated. In Figure 5b, the nozzle path, including the pattern, the main sample, and the transition walls (outer wall) formed between the pattern and the base material, is shown on the five-pattern specimen. When a pattern is placed on the surface of the specimen, the nozzle path changes according to the shape of the pattern. It was observed that two walls were formed during the transition from the pattern to the base material. The continuity of the material is interrupted at the transitions between the pattern and the base material. As the patterns are positioned closer to each other, the nozzle movement becomes more difficult, and printing errors increase. In addition, the bonding capability between the pattern and the base material during printing directly affects the strength. In Figure 5c, areas numbered 1 and 2 indicate the narrowed regions on the pattern.

**Figure 6:**
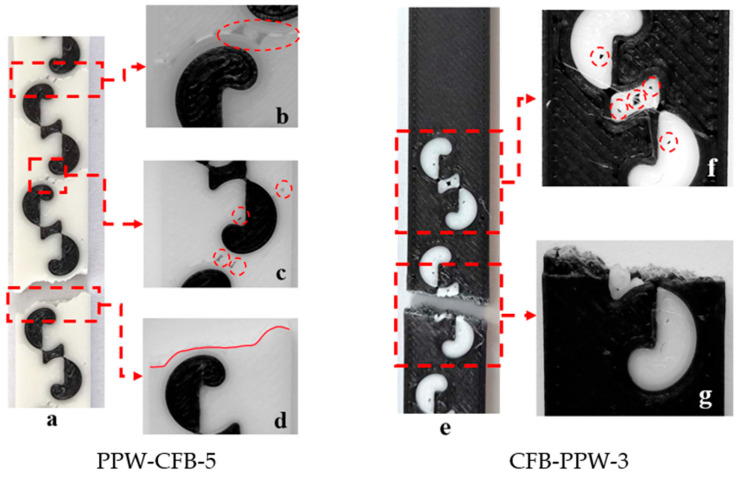
(**a**–**g**) Images of specimens.

Images taken from bending samples with five and three patterns (PPW-CFB-5 and CFB-PPW-3) consisting of PLA Plus (white) and PLA CF materials are given in Figure 6. Figure 6a shows the five-patterned PLA Plus base material and the PLA CF patterned material (PPW-CFB-5), while Figure 6e shows the three-patterned PLA CF base material and the patterned material PLA Plus (CFB-PPW-3). Figure 6a,b show the defects that occurred during printing and the fracture regions observed during the bending tests. As the number of patterns on the specimen increased and the patterns became closer to each other, voids formed around the outer walls (Figure 6a–c). In Figure 6a, it can be seen that during the bending test, the specimen was stressed at the outer walls surrounding the patterns, and fractures occurred in these regions (Figure 6d). In Figure 6f, a void formed in the central part of the pattern during printing is visible. Figure 6g shows the fracture regions of the CFB-PPW-3 specimen during bending loading. It was determined that the fracture occurred in the central region of the pattern.

**Figure 7:**
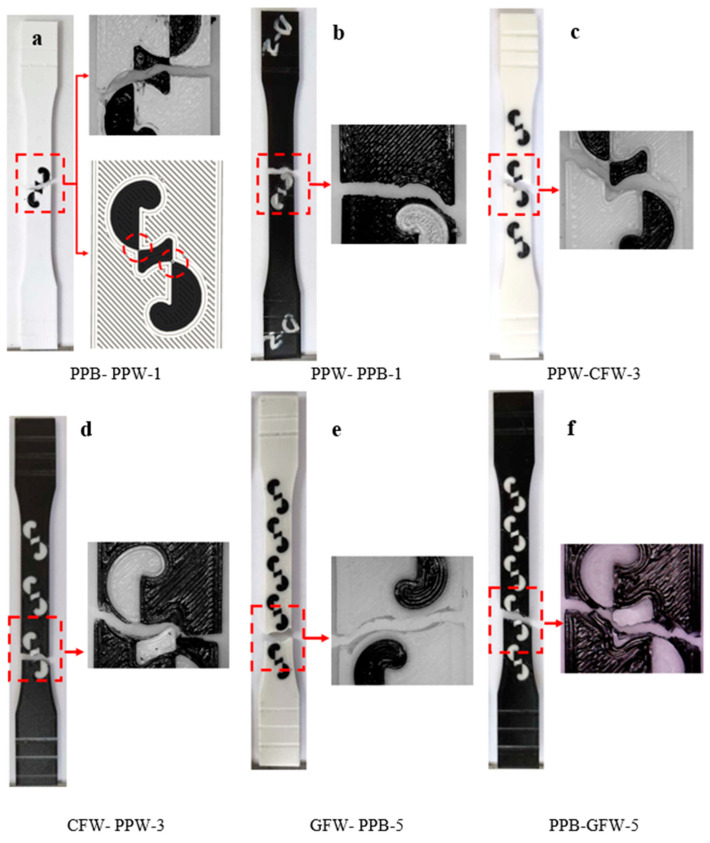
(**a**–**f**) Images of the selected specimens after tensile testing.

Figure 7 shows the regions where fractures occurred after the tensile tests. From each material pair, representative samples were selected: a single-pattern specimen (PLA Plus–PLA Plus), a three-pattern specimen (PLA Plus–PLA CF), and a five-pattern specimen (PLA Plus–PLA GF). Since the pattern dimensions on the tensile specimens were relatively larger compared to those on the bending specimens, printing defects were reduced.

In Figure 7a,b (single-pattern specimens), two different fracture modes were observed. In Figure 7a, the fracture occurred through the pattern, whereas in Figure 7b, the fracture followed the outer walls surrounding the pattern. The fracture mode observed in Figure 7a is thought to originate from the weak regions at the pattern transitions. These weak transition zones are indicated in Figure 7a. In the three-pattern specimens, fractures occurred through the patterns, while in the five-pattern specimens, fractures occurred both through the pattern and by following the outer wall (Figure 7c–f).

**Figure 8:**
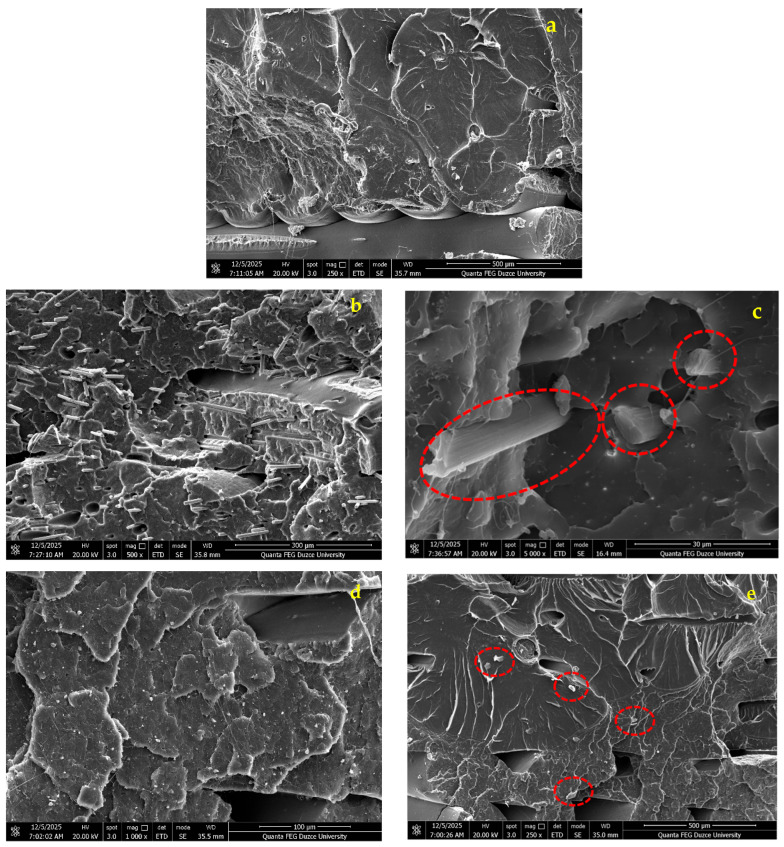
(**a**–**e**) PLA Plus, appearance of carbon fiber and glass fiber additives.

Figure 8 presents the SEM images of the fracture surfaces of the specimens. Figure 8a shows the microstructural images of PLA Plus, Figure 8b,c show those of PLA CF, and Figure 8d,e show those of PLA GF. Fractured CF fibers can be observed in Figure 8b,c. The lengths of the broken CF fibers may differ from one another. This is due to excessive shear forces acting on the fibers, either caused by the movement of the print head [41,42] or by the application of an external load [43]. It is seen that the CF fibers are embedded in the PLA matrix and surrounded by PLA [44]. In addition, voids resulting from the breakage of CF fibers are visible in Figure 8b. In Figure 8d,e, the distribution of the glass fiber reinforcement within the polymer matrix is relatively uniform, without any agglomeration. The glass fiber reinforcements are also seen to be surrounded by PLA.

**Figure 9:**
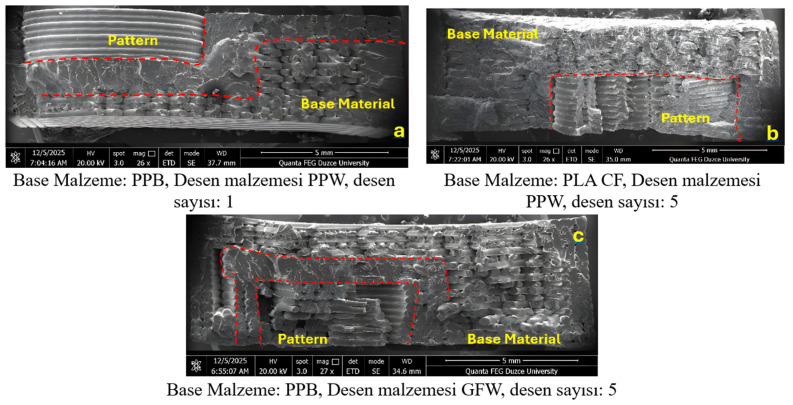
(**a**–**c**) Images of fractured surfaces.

Figure 9 presents the fracture surface images of the patterned specimens with the highest tensile strength (a: PPB-PPW-1, b: CFB-PPW-5, c: PPB-GFW-5). The pattern and base-material regions are marked on the images. In Figure 9a, since the fracture occurred through the wall, no deformation was observed in the patterned region. However, it was observed that the fracture continued around the patterned region, resulting in smooth, laminated fracture surfaces. As the distance from the pattern increases, the printing layers in the part become more visible.

Since the base material in Figure 9b is PLA CF, irregular and rough surfaces exhibiting ductile fracture characteristics formed in the region outside the pattern due to the effect of the CF fibers [45]. In Figure 9c, the fracture occurred from the pattern. The fracture took place in a narrower area compared with the PLA CF material shown in Figure 9b. This indicates that the GF fibers exhibit a weaker bonding with the PLA matrix.

### 3.3. Tensile Test Results

#### 3.3.1. PLA Plus-PLA Plus Tensile Test Results

**Figure 10:**
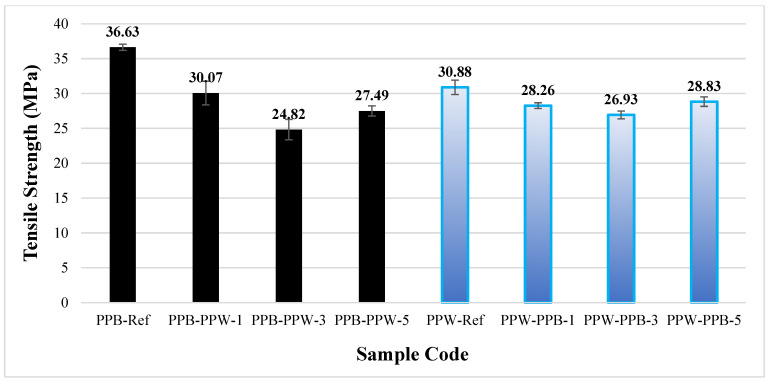
Tensile strengths of unpatterned (Ref) and patterned PLA Plus samples.

Figure 10 presents the tensile strengths of the pattern-free (Ref) and patterned PLA Plus samples (in black and white). The strength of the black PLA Plus reference specimens was determined to be 36.63 MPa, while that of the white PLA Plus reference specimens was 30.88 MPa. The difference in strength between the black and white PLA Plus samples can be attributed to the color pigments added to the filaments by the manufacturer [35,36].

When the patterned and reference specimens were compared within their respective color groups, all patterned samples exhibited lower tensile stresses than the reference samples. This can be attributed to the interface between the pattern and the base material. In the specimens where the base material was black PLA Plus and the pattern was white PLA Plus, the closest tensile strength to the reference value was obtained in the single-pattern specimen at 30.07 MPa, while the lowest tensile strength was found in the three-pattern specimen at 24.82 MPa (a 32.24% decrease compared to the reference).

In the specimens where the base material was white PLA Plus and the pattern was black PLA Plus, the tensile strength values of the samples with 1, 3, and 5 patterns were 28.26, 26.93, and 28.83 MPa, respectively, showing relatively similar results. More consistent results were obtained in the specimens where the pattern was printed using black PLA Plus. When the tensile strength of the white PLA Plus reference specimen is compared with that of the three-pattern specimen, which exhibited the lowest strength, a decrease of 13.11% is observed.

Table 4 presents the strength, percentage elongation, and Young’s modulus values for all specimens. A decrease was observed in all patterned samples compared to the reference specimens. This decrease is attributed to crack initiation occurring at the interface between the pattern and the base material. Examination of the fracture surfaces after the tensile test showed that the failure occurred through the patterned region. It was also determined that the Young’s modulus increased as the number of patterns in the specimens increased.

**Figure 11:**
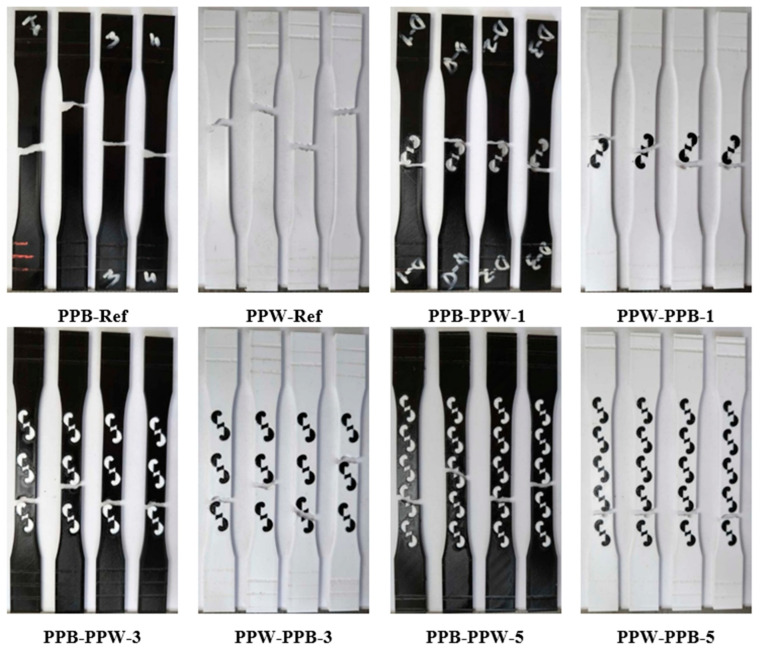
Fracture surfaces of PLA Plus-PLA Plus samples.

Figure 11 shows the fracture surfaces of the PLA Plus–PLA Plus specimens after the tensile tests. When the PLA Plus reference samples are compared with each other, their fracture patterns appear similar despite the difference in color. In the patterned samples, the fractures occurred along the pattern boundary line for both colors.

#### 3.3.2. PLA Plus—PLA CF Tensile Test Results

**Figure 12:**
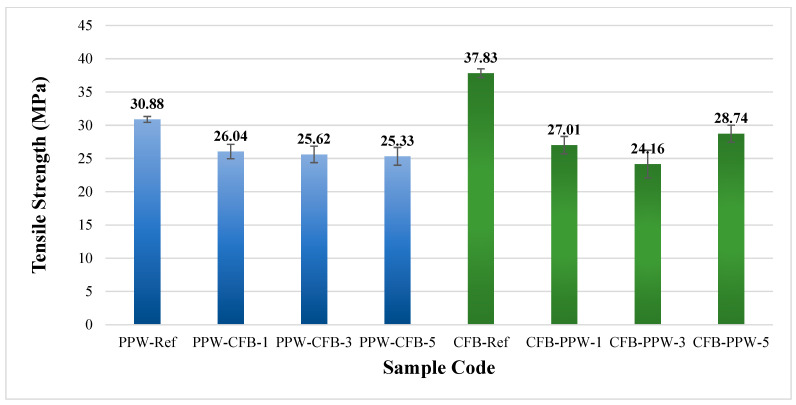
Tensile strengths of unpatterned (Ref) and patterned PLA Plus and PLA CF samples.

Figure 12 presents the tensile strengths of the patterned and unpatterned samples of white PLA Plus and black PLA CF. The tensile strength was determined as 30.88 MPa for the white PLA Plus reference sample and 37.83 MPa for the black PLA CF reference sample. Pazhamannil et al. [45] investigated the effects of infill ratio, infill pattern, layer height, extruder temperature, and print speed on strength using Raise3D Premium PLA (Raise3D, Stafford, TX, USA) and Sunlu PLA Carbon Fiber thermoplastic filaments (SUNLU, Montauk, NY, USA). They reported tensile strength values of 31.11 MPa for PLA and 37.27 MPa for PLA CF when prints were produced using optimized process parameters. The increase in strength observed in PLA CF was explained by the reinforcing effect of carbon fibers, which act as a frame structure and promote heterogeneous nucleation [46]. The results obtained in the present study are consistent with and support the tensile strength values reported in previous work for PLA and PLA CF (Table 5).

**Figure 13:**
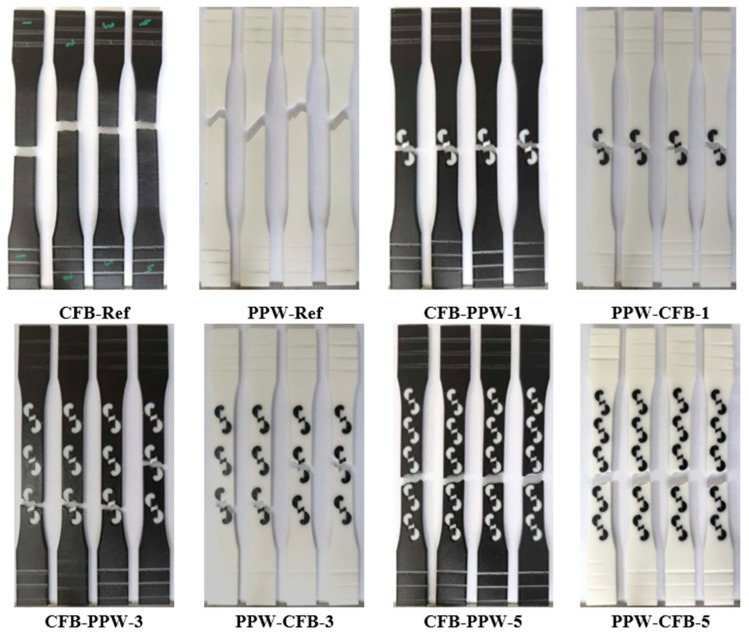
Fracture surfaces of PLA Plus-PLA CF samples after tensile testing.

Figure 13 shows the fracture surfaces of the PLA Plus–PLA CF specimens after the tensile tests. When the reference samples are compared, it is observed that the PLA Plus specimens fractured at an angle relative to the loading direction, whereas the PLA CF specimens fractured perpendicularly in a straight manner. In the single-pattern and three-pattern specimens, the applied load propagated through the central region of the pattern, leading to failure of the specimens. In the five-pattern specimens, however, the fracture followed the boundary lines of the pattern.

#### 3.3.3. PLA Plus-PLA GF Tensile Test Results

**Figure 14:**
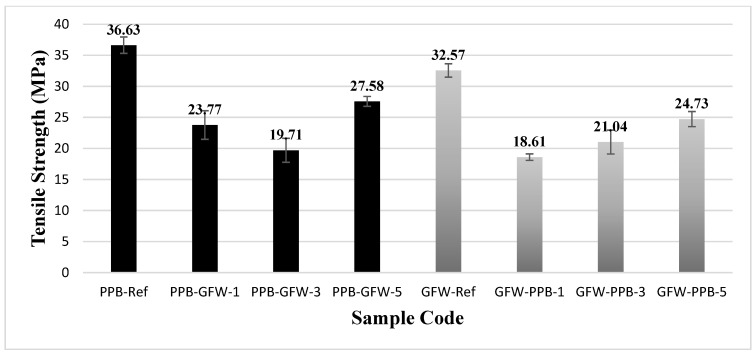
Tensile strengths of unpatterned (Ref) and patterned PLA Plus and PLA GF samples.

Figure 14 presents the tensile strengths of the patterned and unpatterned specimens of black PLA Plus and PLA GF. The tensile strength was determined as 36.63 MPa for the PLA Plus reference specimens and 32.57 MPa for the PLA GF reference specimens. Gain and Zhang used a 5% glass fiber reinforcement to improve the mechanical properties of pure PLA and PEEK for orthopedic applications. They reported that the strength of PLA increased from 22.9 MPa to 30.4 MPa, and the strength of PEEK increased from 61.3 MPa to 103.08 MPa [47]. Wang et al. added glass fiber to PLA at four different ratios (5%, 10%, 15%, and 20%). They stated that as the GF content increased, the tensile strength, tensile modulus, and impact strength also increased, while the percentage elongation decreased [48]. However, it is well known that in fiber-reinforced materials, factors such as fiber volume fraction, fiber length and shape, matrix type, and production parameters significantly influence the mechanical properties. Therefore, the differences between the results obtained from commercial filaments and those reported by researchers who produce their own filaments are considered acceptable.

When all combinations were evaluated, the greatest reductions in tensile strength were observed in the three-pattern specimen with PLA Plus as the base material and PLA GF as the pattern material (PPB-GFW-3), with a decrease of 46.2%, and in the single-pattern specimen with PLA GF as the base material and PLA Plus as the pattern material (GFW-PPB-1), with a decrease of 42.8% (Table 6).

**Figure 15:**
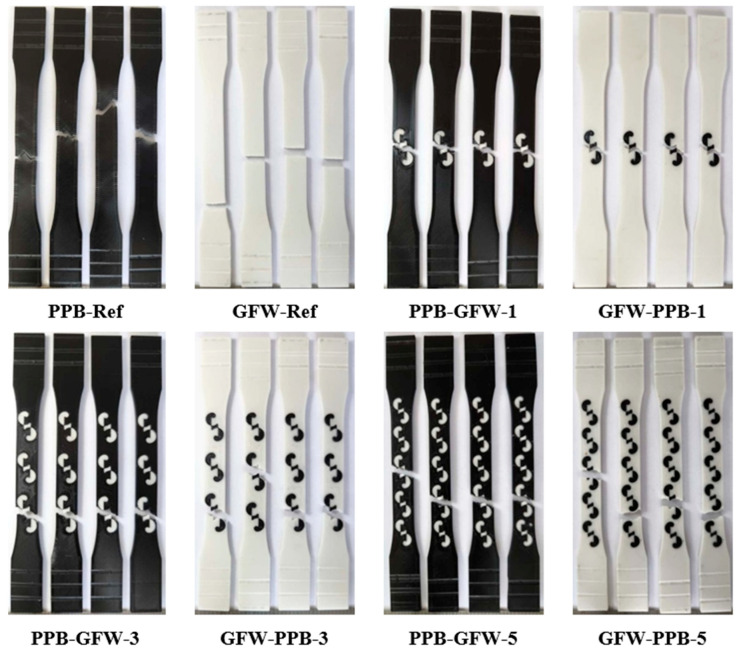
Fracture surfaces of PLA Plus-PLA GF samples after tensile testing.

Figure 15 shows the fracture surfaces of the PLA Plus–PLA GF specimens after the tensile tests. Examination of the reference samples reveals that the fracture appearance of the PLA GF specimens resembles that of the PLA CF reference specimens shown in Figure 12. In all patterned samples, failure occurred as the fracture propagated through the central region of the pattern.

### 3.4. Bending Test Results

#### 3.4.1. PLA Plus-PLA Plus Bending Test Results

**Figure 16:**
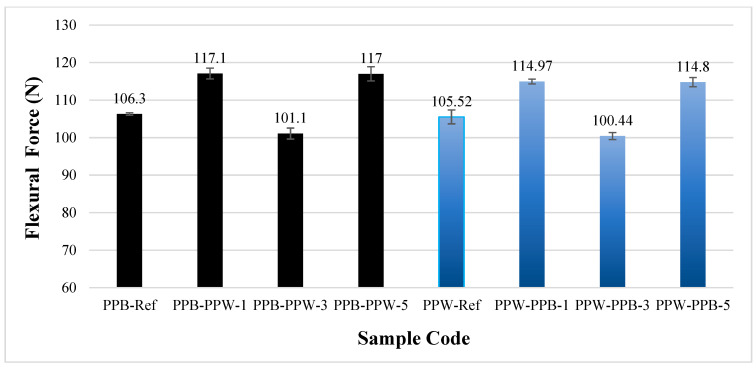
Bending force of unpatterned (Ref) and patterned PLA Plus (black and blue) samples.

Figure 16 presents the bending force obtained from the bending tests for the pattern-free (Ref) and patterned PLA Plus samples (in black and white). The bending force was determined as 106.3 ± 0.3 N for the black reference specimens and 105.52 ± 1.8 N for the white reference specimens. The bending test results indicate that color had a minimal effect (0.73%) on the bending force of the reference samples. In the three-pattern specimens of both color combinations, the bending force decreased (101.1 ± 1.4 N and 100.4 ± 0.9 N, respectively). In the single-pattern specimens with black as the base material and white as the pattern material, the bending force increased to 117.1 ± 1.4 N (a 10.1% increase), while in the five-pattern specimens it increased to 117 ± 1.9 N (a 10% increase). In the specimens with white as the base material and black as the pattern material, the bending force reached 114.97 ± 0.6 N (an 8.9% increase) in the single-pattern samples and 114.80 ± 1.2 N (an 8.7% increase) in the five-pattern samples. According to the bending test results, incorporating one or five patterns increased the bending force in both color combinations. However, no significant difference was observed between the bending force of the single-pattern and five-pattern specimens.

**Figure 17:**
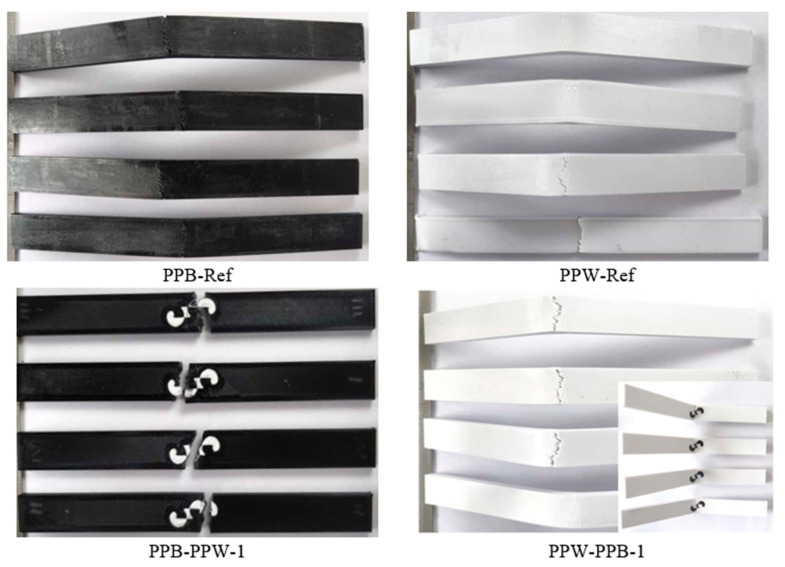
Images of PLA Plus-PLA Plus reference and single-patterned samples after bending test.

Figure 17 shows the appearance of PLA Plus-PLA Plus reference and single-patterned samples after the bending test. For all fracture surface images, the specimens with the highest bending force were selected. In the single-pattern PPB-PPW-1 coded specimens, the samples completely separated into two parts after the bending test, whereas in the PPW-PPB-1 coded specimens, the applied strength caused cracks to form and bent the part without full separation.

#### 3.4.2. PLA Plus-PLA CF Bending Test Results

**Figure 18:**
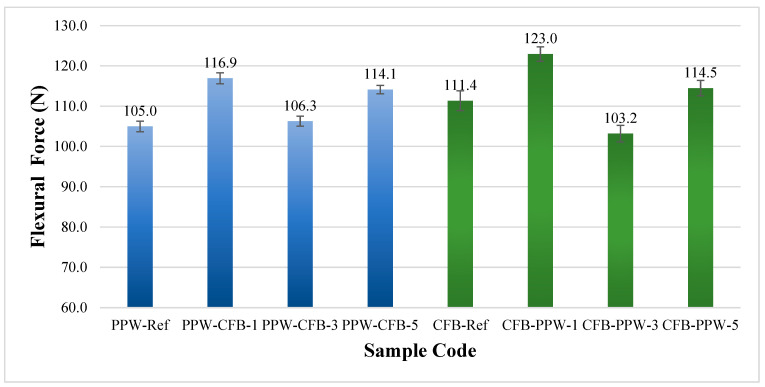
Bending forces of unpatterned (Ref) and patterned PLA Plus and PLA CF samples.

Figure 18 presents the bending forces obtained after the bending tests for the patterned and unpatterned specimens of white PLA Plus and black PLA CF. The bending force was measured as 105.0 ± 2.5 N for the PLA Plus reference specimen and 111.4 ± 1.8 N for the PLA CF reference specimen. The addition of carbon fiber increased the bending force of PLA CF by 6% compared to PLA Plus. Li et al. examined the mechanical properties of different PLA/PLA CF material combinations and reported that specimens produced using filaments containing 15% carbon fiber exhibited higher flexural strength [49]. The findings of the two studies are consistent with each other. In the specimens where PLA Plus was used as the base material and PLA CF as the pattern material, the bending force increased. Compared to the reference PLA Plus specimens, the bending force in the single-, three-, and five-pattern samples (116.9 ± 1.8, 106.3 ± 2.1, and 114.1 ± 1.9 N) increased by 11.3%, 1.2%, and 8.6%, respectively. While the increase in bending force was greater in the single- and five-pattern specimens, the increase was relatively lower in the three-pattern specimens. In the samples where PLA GF was used as the base material and PLA Plus as the pattern material, an increase was observed in the single- and five-pattern specimens, whereas a decrease occurred in the three-pattern specimens. Compared to the reference PLA CF samples, the bending forces of the single-, three-, and five-pattern specimens (123.0 ± 1.4, 103.2 ± 1.2, and 114.5 ± 1.0 N) changed by 10.4%, −7.3%, and 2.7%, respectively.

**Figure 19:**
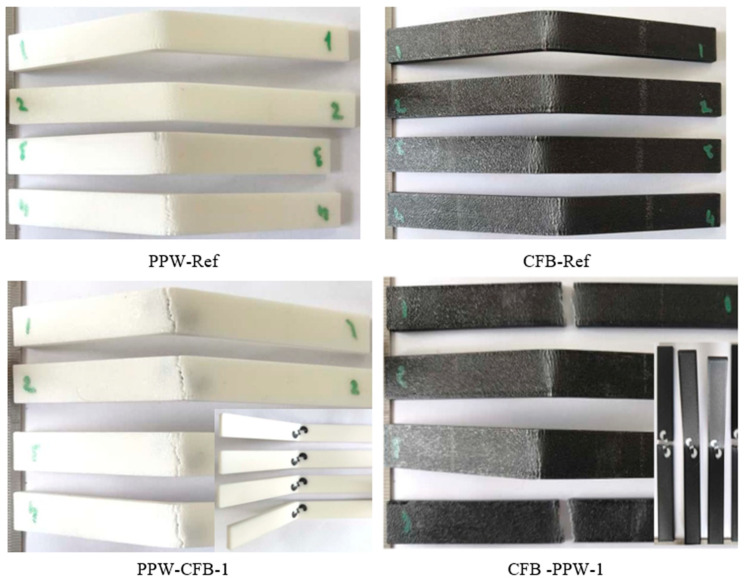
Images of PLA Plus-PLA CF reference and single-patterned samples after bending test.

Figure 19 shows the fracture surfaces of the PLA Plus–PLA CF reference and single-pattern specimens after the bending test. When the reference samples are compared, it is observed that although the materials differ, the bending behavior is similar in form. Additionally, in the bending specimens, the load-induced lines are visibly concentrated in the fracture region in the form of cracks.

#### 3.4.3. PLA Plus-PLA GF Bending Test Results

**Figure 20:**
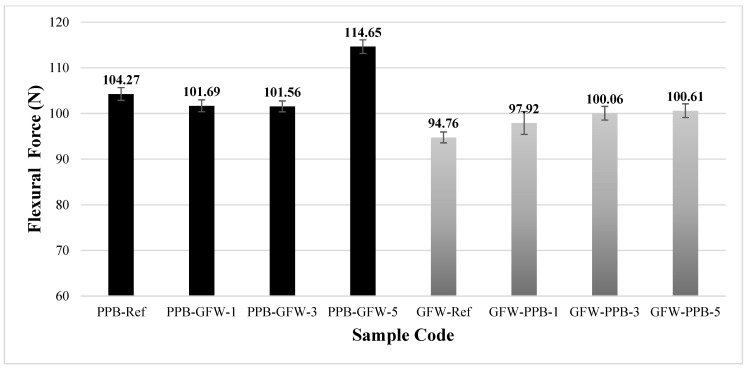
Bending forces of unpatterned (Ref) and patterned PLA Plus and PLA GF samples.

Figure 20 presents the bending forces obtained after the bending tests for the patterned and unpatterned specimens of black PLA Plus and white PLA GF. The bending force was measured as 104.27 ± 1.4 N for the PLA Plus reference specimen and 94.76 ± 1.2 N for the PLA GF reference specimen. In the reference samples, the addition of glass fiber resulted in a 9.12% decrease in bending force. Magalhães et al. investigated the post-printing mechanical properties, thermal stability, and fracture behavior of PLA, PLA CF, and PLA GF specimens. They reported that the presence of fibers prevents the material from being fully compacted during printing and that adding fiber reinforcement to the filament increases the void percentage [50]. Depending on the characteristics of the fibers added to the filament, the mechanical properties can vary significantly. In the specimens where PLA Plus was used as the base material and PLA GF as the pattern material, the bending force decreased by −2.4% (101.69 N) in the single-pattern sample and −2.5% (101.56 N) in the three-pattern sample. In the five-pattern specimen, however, the bending force increased by 9.9% (114.65 N). In the specimens where PLA GF was used as the base material and PLA Plus as the pattern material, an increase in bending force was observed in all single-, three-, and five-pattern samples. Compared to the pattern-free (reference) PLA GF specimen, the bending force values for the single-, three-, and five-pattern samples (97.92 ± 12.5, 100.06 ± 1.5, and 100.61 ± 1.5 N) changed by 3.3%, 5.5%, and 6.1%, respectively.

**Figure 21:**
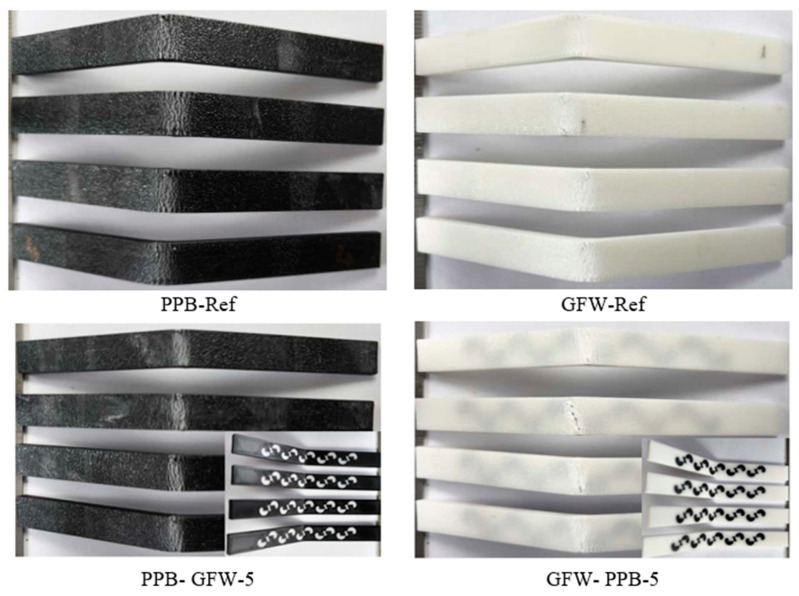
Images of PLA Plus-PLA GF reference and five-patterned samples after bending test.

Figure 21 shows the post-bending test images of the PLA Plus–PLA GF reference and five-pattern specimens. After the bending test, it was observed that the specimens bent at the point of load application but did not fully fracture. The cracks formed on the surface were observed to follow a straight line extending across the width of the specimens.

## 4. Conclusions and Recommendations

When the results of this study are examined, it is observed that tensile strength generally decreased in the patterned specimens. The decrease in tensile strength in the patterned parts is attributed to the outer walls surrounding the patterns, which adversely affect the structural integrity of the part.

The bending test results showed that the bending force increased in the single-pattern and five-pattern specimens compared to the reference samples. This increase is thought to result from the compression of both the pattern material and the base material during bending. In the black–white PLA Plus combination (where the black filament was used as the base material and the white filament as the pattern), the bending force of the PPB-PPW-1 specimen increased by 10.16% compared to the reference sample. In the PLA Plus–PLA CF combination, when PLA Plus was used as the base material, the bending force increased by 11% in the PPW-CFB-1 specimen; when PLA CF was used as the base material, the bending force increased by 10.4% in the CFB-PPW-1 specimen. In the PLA Plus–PLA GF combination, when PLA Plus was used as the base material, the bending force increased by 9.9% in the PPB-GFW-5 specimen, and when PLA GF was used as the base material, it increased by 6.1% in the GFW-PPB-5 specimen. These findings indicate that when white PLA Plus, PLA CF, or PLA GF is used as the pattern material, the increase in bending force is more pronounced. Printing parts composed of different materials together as a single piece on the same 3D printer build plate provide significant advantages for manufacturers, such as reduced cost and time. In this study, multi-material printing was achieved by adding a designed pattern onto the part surface using a 3D printer. Since most natural patterns have indistinct boundaries, transferring pattern information into a digital model is not straightforward. However, with the use of advanced artificial intelligence applications in future studies, it may become possible to analyze natural patterns in greater detail and represent them more clearly. This would enable the direct transfer of natural patterns onto part surfaces in an identical form. Consequently, beyond the currently limited use of biomimetic applications, it is believed that we may be able to benefit from the possibilities offered by nature in their most authentic form. Additionally, with the continuous advancement of 3D printing technology, achieving more precise prints through the use of smaller nozzle diameters and reduced layer heights will further facilitate patterned part production.

## Figures and Tables

**Table 1:** Mechanical Properties of Materials.

Mechanical Properties	PLA Plus [31]	PLA GF [32]	PLA CF [33]
Density (g/cm^3^)	1.23	1.31	1.21
Tensile Strength (MPa)	53.34	59.27	59.64
Tensile Elongation (%)	20	7.99	5.26
Flexural Strength (MPa)	81.16	85.01	103

**Table 2:** Sample coding.

Main (Base) Material	Main (Base) Material Color	Pattern Material	Pattern Color	Pattern Number	Code
PLA Plus	Black	PLA Plus	White	1	PPB-PPW-1
PLA Plus	Black	PLA GF	White	3	PPB-GFW-3
PLA Plus	White	PLA CF	Black	5	PPW-CFB-5
PLA GF	White	PLA Plus	Black	3	GFW-PPB-3
PLA CF	Black	PLA Plus	White	3	CFB-PPW-3

**Table 3:** Mechanical properties of reference samples.

	Material	UTS (MPa)	Elongation %	Modulus of Elasticity (MPa)		Bending Force (N)
Tensile Test	PLA Plus Black	36.63 ± 0.4	6.75	572.7	Bending Test	106.3 ± 0.3
	PLA Plus White	30.80 ± 1.0	4.82	836.4		105.5 ± 0.6
	PLA CF	37.83 ± 0.4	9.23	1077.06		111.4 ± 1.3
	PLA GF	32.57 ± 1.1	3.30	1077.00		94.7 ± 1.2

**Table 4:** Mechanical properties of PLA Plus-PLA Plus patterned samples.

Sample Code	Tensile Strength (MPa)	Elongation (%)	Young’s Modulus (MPa)
PPB-Ref	36.63 ± 0.4	6.75 ± 0.4	572.7 ± 37.4
PPB-PPW-1	30.07 ± 1.6	2.86 ± 0.2	610.2 ± 39.3
PPB-PPW-3	24.82 ± 1.4	2.58 ± 0.2	1019.6 ± 98.6
PPB-PPW-5	27.49 ± 0.7	3.01 ± 0.2	1030.9 ± 63.1
PPW-Ref	30.88 ± 1.0	4.82 ± 0.5	836.4 ± 60.2
PPW-PPB-1	28.26 ± 0.4	1.62 ± 0.1	874.9 ± 77.9
PPW-PPB-3	26.93 ± 0.5	2.41 ± 0.1	801.6 ± 95.1
PPW-PPB-5	28.83 ± 0.6	2.84 ± 0.2	1058.1 ± 65.5

**Table 5:** Mechanical properties of PLA Plus-PLA CF samples.

Sample Code	Tensile Strength (MPa)	Elongation (%)	Young’s Modulus (MPa)
**PPW-Ref**	30.88 ± 1.08	7.6 ± 1.0	1077.06 ± 121
**PPW-CFB-1**	26.04 ± 1.34	2.4 ± 0.05	1126.44 ± 140
**PPW-CFB-3**	25.62 ± 1.31	2.85 ± 0.14	1156.07 ± 86
**PPW-CFB-5**	25.33 ± 1.28	2.68 ± 0.17	1105.69 ± 16
**CFB-Ref**	37.83 ± 0.45	9.23 ± 1.1	1077.06 ± 105
**CFB-PPW-1**	27.01 ± 1.08	2.2 ± 0.08	1178.74 ± 158
**CFB-PPW-3**	24.16 ± 0.66	2.6 ± 0.02	814.56 ± 51
**CFB-PPW-5**	28.74 ± 2.09	2.4 ± 0.12	1143.0 ± 89

**Table 6:** Mechanical properties of PLA Plus-PLA GF patterned samples.

Sample Code	Tensile Strength (MPa)	Elongation (%)	Young’s Modulus (MPa)
PPB-Ref	36.63 ± 1.3	9.6 ± 1.8	902.8 ± 46
PPB-GFW-1	23.77 ± 2.3	2.3 ± 0.04	945.5 ± 125
PPB-GFW-3	19.70 ± 1.9	2.2 ± 0.1	912.7 ± 126
PPB-GFW-5	27.57 ± 0.8	2.9 ± 0.1	912.7 ± 187
GFW-Ref	32.57 ± 1.1	3.3 ± 0.1	1077.0 ± 121
GFW-PPB-1	18.60 ± 0.5	1.4 ± 0.03	1361.7 ± 125
GFW-PPB-3	21.03 ± 1.9	1.4 ± 0.1	912.7 ± 164
GFW-PPB-5	24.73 ± 1.2	1.7 ± 0.1	1027.9 ± 110

## Data Availability

The original contributions presented in this study are included in the article. Further inquiries can be directed to the corresponding author.
